# Reticulocyte count: a simple test but tricky interpretation!

**DOI:** 10.11604/pamj.2021.40.3.31316

**Published:** 2021-09-02

**Authors:** Malvika Gaur, Tushar Sehgal

**Affiliations:** 1 Department of Laboratory Medicine, All India Institute of Medical Sciences, New Delhi, India

**Keywords:** Corrected reticulocyte count, reticulocyte index, reticulocyte proliferation index

## To the editors of the Pan African Medical Journal

International Council for Standardization in Hematology (ICSH) defines reticulocytes as non-nucleated red blood cells (RBC) that contain at least two blue staining particles or one particle linked to a filamentous thread [1]. The name reticulocyte comes from reticular or mesh-like network. This reticular network is composed of ribosomal ribonucleic acid (RNA) that becomes visible under a light microscope by certain dyes such as brilliant cresyl blue and new methylene blue. As the cells are still in a living state when exposed to the dye, this is referred to as supravital staining [2]. Reticulocytes are classified into four groups, ranging from the most immature with a large clump of reticulin (group I), to the most mature, with a few granules of reticulin (group IV) [2].

### Why is it important to understand and study about reticulocytes?

The reticulocytes are an important guide of the amount of RBC produced in bone marrow and entering the peripheral blood. It is therefore an index of effective erythropoiesis. Its normal fraction in the blood is low (0.5% to 2.5% in adults and 2% to 6% in infants) because there is a homeostasis between destruction of aged abnormal RBC and a low level of marrow activity required to maintain normal hemoglobin levels [3].

### How should a reticulocyte be ´correctly´ reported?

Most mature reticulocytes contain only a few dots of reticulofilamentous material hence the interpretation may be tricky. Moreover, it may sometimes be difficult to differentiate from other RBC inclusions such as Pappenheimer bodies, Howell-Jolly bodies, Heinz bodies or Hemoglobin H inclusions [2]. To address this problem and to accurately measure reticulocyte, automated hematology analyzers use a combination of laser excitation and fluorescence dyes (such as thiazole orange or polymethine) that label reticulocyte RNA [4]. The number of reticulocytes in the peripheral blood is an accurate reflection of erythropoietic activity. However, if the patient has moderate or severe anemia, the bone marrow will release reticulocytes prematurely into the blood. These prematurely-released reticulocytes are called “shift reticulocytes”, and they will circulate in the peripheral blood for longer than normally-released reticulocytes. In such case a laboratory physician must give a corrected count expressed as reticulocyte index (RI) or reticulocyte proliferation index (RPI) to avoid spurious results [3,5]. Their formulae are as follows: a) RI=observed reticulocyte [%] x patient´s hemoglobin or hematocrit/standard hemoglobin or hematocrit; b) RPI= RI x (1/reticulocyte maturation time in days).

**Figure 1:**
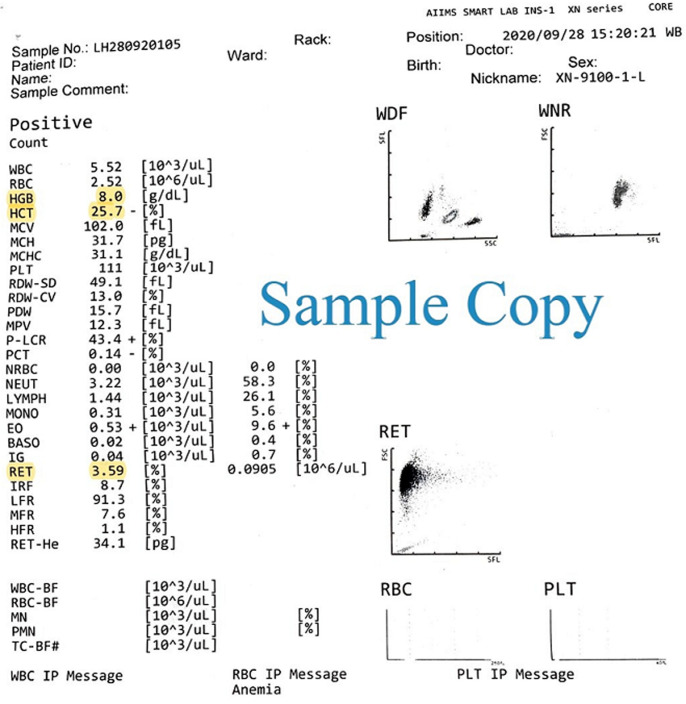
a sample haemogram report of an adult male from an automated hematology analyser, the uncorrected reticulocyte count was 3.59%, which is higher than the normal range for adults; this may give a false interpretation to the treating clinician of an adequate reticulocyte response from the bone marrow; however, after correction for hematocrit the reticulocyte index (RI) was 2%

Figure 1 shows a sample haemogram report of an adult male from an automated hematology analyzer. The uncorrected reticulocyte count was 3.59%, which is higher than the normal range for adults. This may give a false interpretation to the treating clinician of an adequate reticulocyte response from the bone marrow. However, after correction for hematocrit the RI was 2% which is in the normal range.

**Interpretation:** RI <2% with anemia indicates decreased production of reticulocytes (i.e. inadequate response to correct the anemia) and therefore RBCs. RI >3% with anemia indicates loss of RBCs (from causes such as hemolysis, bleeding etc.) with an increased compensatory production of reticulocytes to replace the lost RBC [3].

**Conclusions:** the reticulocyte count serves as a key tool to assess the bone marrow´s ability to increase RBC production in response to various types of anaemias. Precise and accurate reporting of reticulocyte count is imperative as raw reticulocyte count may be misleading in anaemic patients.
